# Supplementary material for: Suppressing MTERF3 inhibits proliferation of human hepatocellular carcinoma via ROS-mediated p38 MAPK activation
Source: Commun Biol. 2024 Jan 5;7:18. doi: 10.1038/s42003-023-05664-7 (PMC10767110; doi:10.1038/s42003-023-05664-7)
Supplement: Supplementary file 2 — Supplementary Information [file 42003_2023_5664_MOESM2_ESM.pdf]

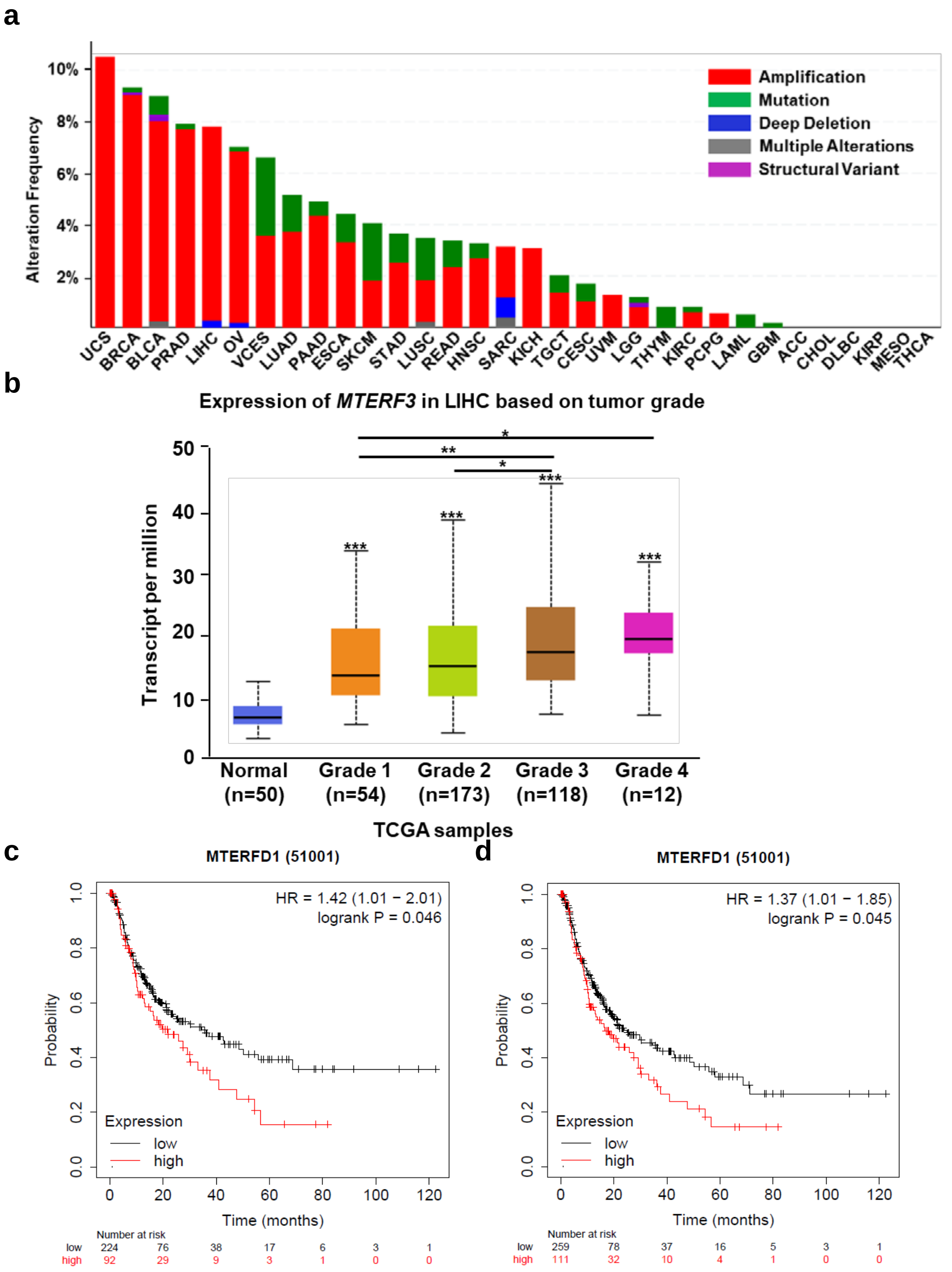

Supplementary Fig. 1 MTERF3 is frequently amplified and upregulated in HCC samples and negatively correlated with prognosis of HCC patients. (a) Using TCGA data to analyze the aberration frequency of *MTERF3* gene in different cancer types. (b) Using TCGA data to analyze the expression of *MTERF3* mRNA in different stages of HCC patients. TCGA data analysis showed that MTERF3 expression is positively correlated with poorer relapse-free survival (c) and progression-free survival (d) of HCC patients. Log-rank test for survival analysis, Student's *t* test; **P*<0.05, ***P*<0.01 and ****P*<0.001.

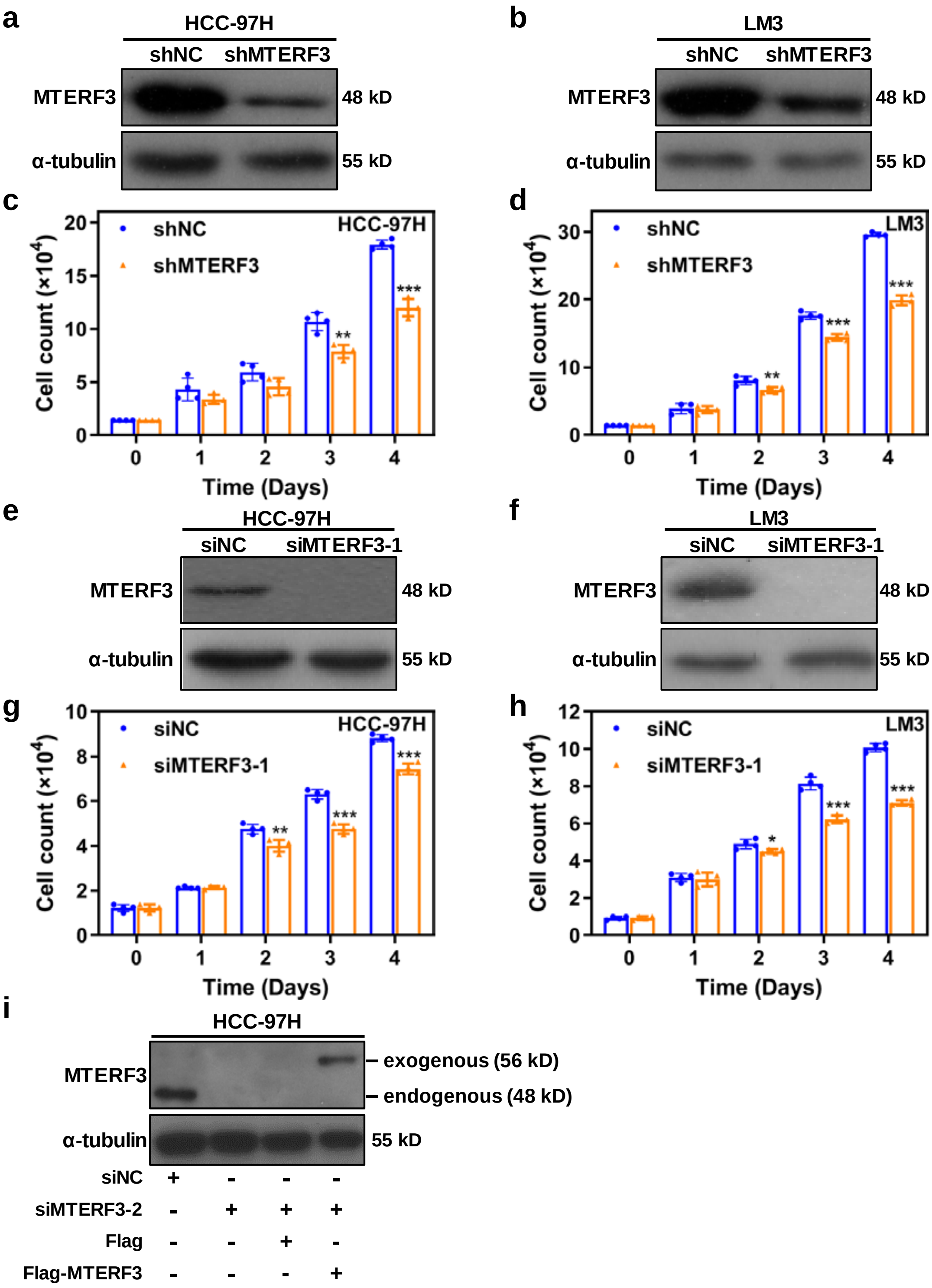

Supplementary Fig. 2 MTERF3 knockdown inhibits cell proliferation of HCC cells. Western blot analysis for MTERF3 expression in HCC-97H (a) or LM3 (b) cells with stably MTERF3 knockdown and controls. Cell proliferation of HCC-97H (c) or LM3 (d) cells with stably MTERF3 knockdown and controls was examined by cell count (n=4). Western blot analysis for MTERF3 expression in HCC-97H (e) or LM3 (f) cells after siMTERF3-1

transfection. Cell proliferation of HCC-97H (g) or LM3 (h) cells after siMTERF3-1 transfection was examined by cell count (n=4). (i) A siRNA targeting 3’-UTR of *MTERF3* mRNA together with indicated vectors were transfected into HCC-97H cells, and MTERF3 expression was analyzed. The experiments were repeated three times independently. Data are shown as mean ± standard deviations of at least three independent experiments. Student’s *t* test; **P*<0.05, ***P*<0.01 and ****P*<0.001.

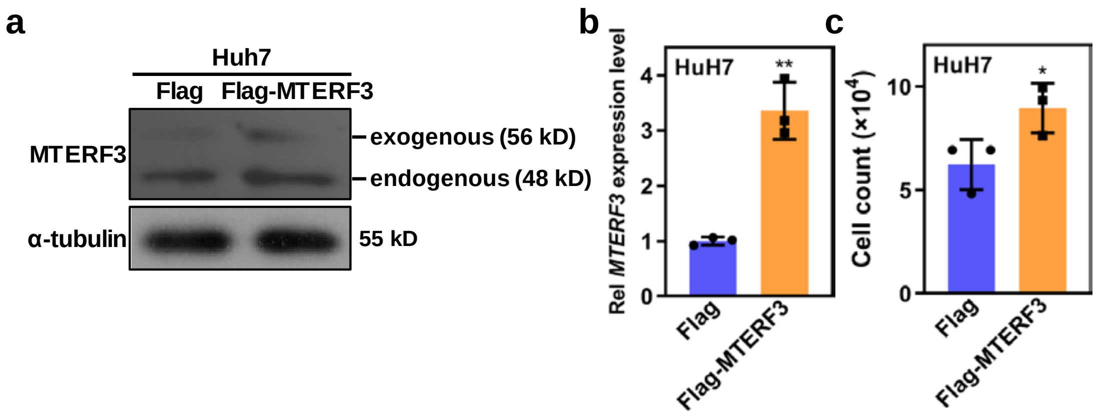

Supplementary Fig. 3 MTERF3 overexpression promotes the proliferation of Huh7 cells. Western bolt analysis (a) or qRT-PCR (b) for MTERF3 expression in Huh7 cells with stably MTERF3 overexpression and controls (n=3). (c) Cell count analysis for cell proliferation of MTERF3 overexpressed Huh7 cells and controls (n=3). Data are shown as mean ± standard deviations of three independent experiments. Student’s *t* test; **P*<0.05 and ****P*<0.001.

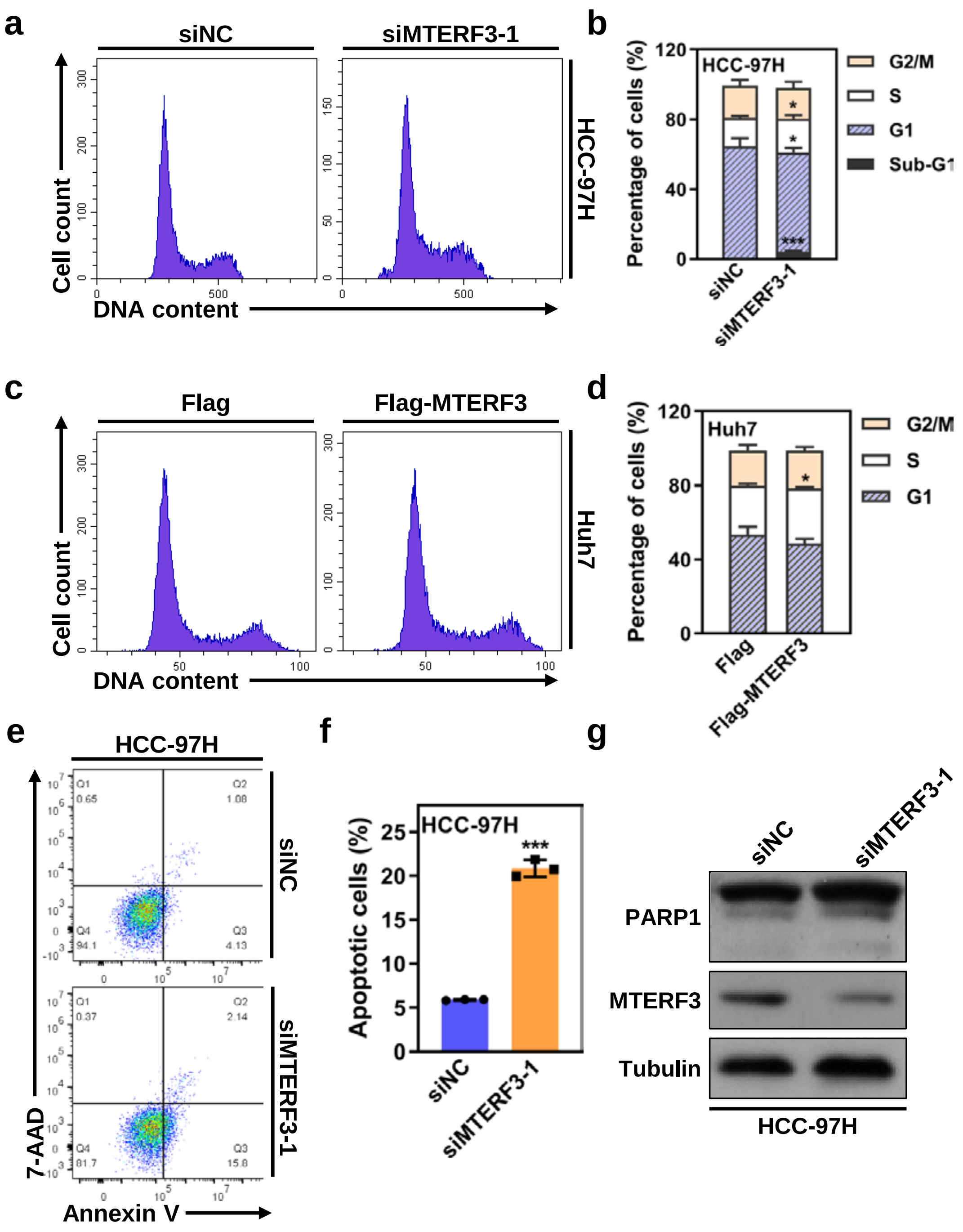

Supplementary Fig. 4 MTERF3 involves in regulating cell cycle progression in HCC cells. (a) Cell cycle profile of HCC-97H cells after transfected with siNC or siMTERF3-1. (b) Cell cycle distribution in (a) was calculated (n=3). (c) Cell cycle profile of Huh7 cells with stably MTERF3 overexpression and controls. (d) Cell cycle distribution in (c) was calculated (n=3). (e) Annexin V/7-AAD staining to analyze cell apoptosis of HCC-97H cells after transfected with siNC or siMTERF3-1. (f) Cell apoptosis in (e) was calculated (n=3). (g) HCC-97H cells were transfected with indicated siRNA for 3 days, and cell lysates were used

to analyze the expression of indicated proteins. Data are shown as mean ± standard deviations of three independent experiments. Student's *t* test; *P<0.05 and ***P<0.001.

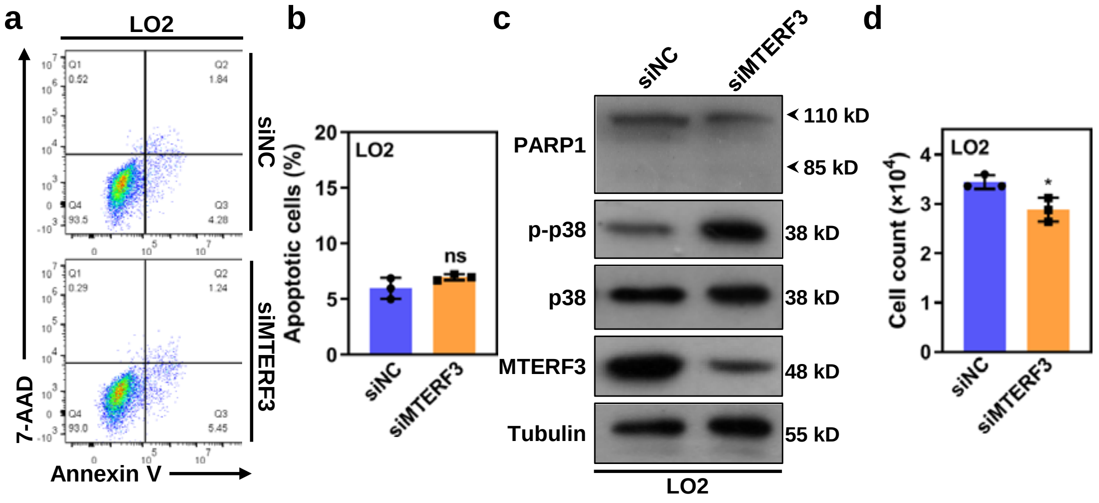

Supplementary Fig. 5 MTERF3 knockdown inhibits cell proliferation but not induces significant apoptosis in normal hepatocytes LO2 cells. (a) Annexin V/7-AAD staining to analyze cell apoptosis of LO2 cells after transfected with siNC or siMTERF3. (b) Cell apoptosis in (a) was calculated (n=3). (c) LO2 cells were transfected with indicated siRNA for 3 days, and cell lysates were used to analyze the expression of indicated proteins. (d) Cell count analysis for cell proliferation of LO2 cells transfected with indicated siRNA for 72 h (n=3). Data are shown as mean ± standard deviations of at least three experiments. Student's *t* test; ns: not significant, *P<0.05.

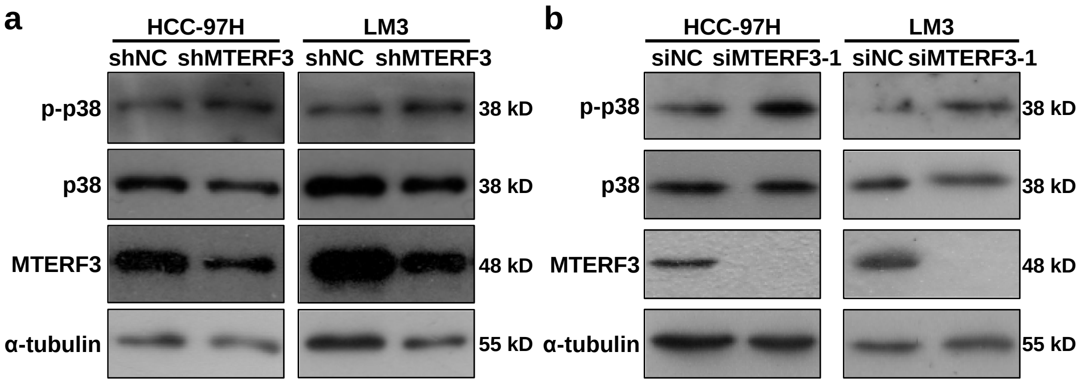

Supplementary Fig. 6 MTERF3 knockdown induces p38 MAPK pathway of HCC cells. (a) Western blot analysis for p38 MAPK activation in LM3 or HCC-97H cells with stably MTERF3 knockdown and controls. (b) Western blot analysis for p38 MAPK activation in LM3 or HCC-97H cells after siMTERF3-1 transfection.

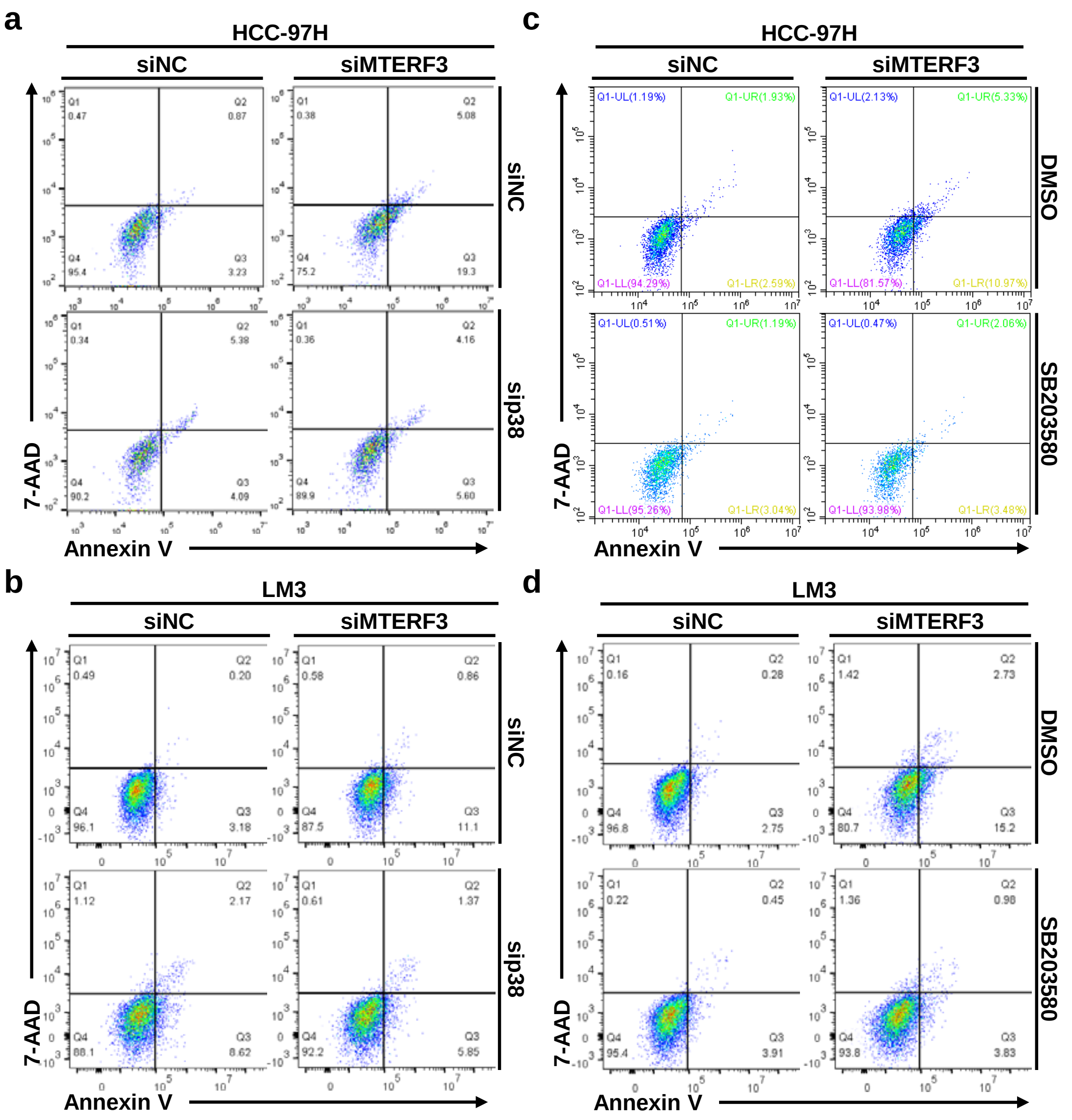

Supplementary Fig. 7 MTERF3 knockdown-induced cell apoptosis depends on p38 MAPK activation. The representative images of Annexin V/7-AAD staining to examine the effects of p38 siRNA transfection (a-b) or p38 inhibitor SB203580 treatment (c-d) on MTERF3-induced apoptosis in HCC-97H or LM3 cells.

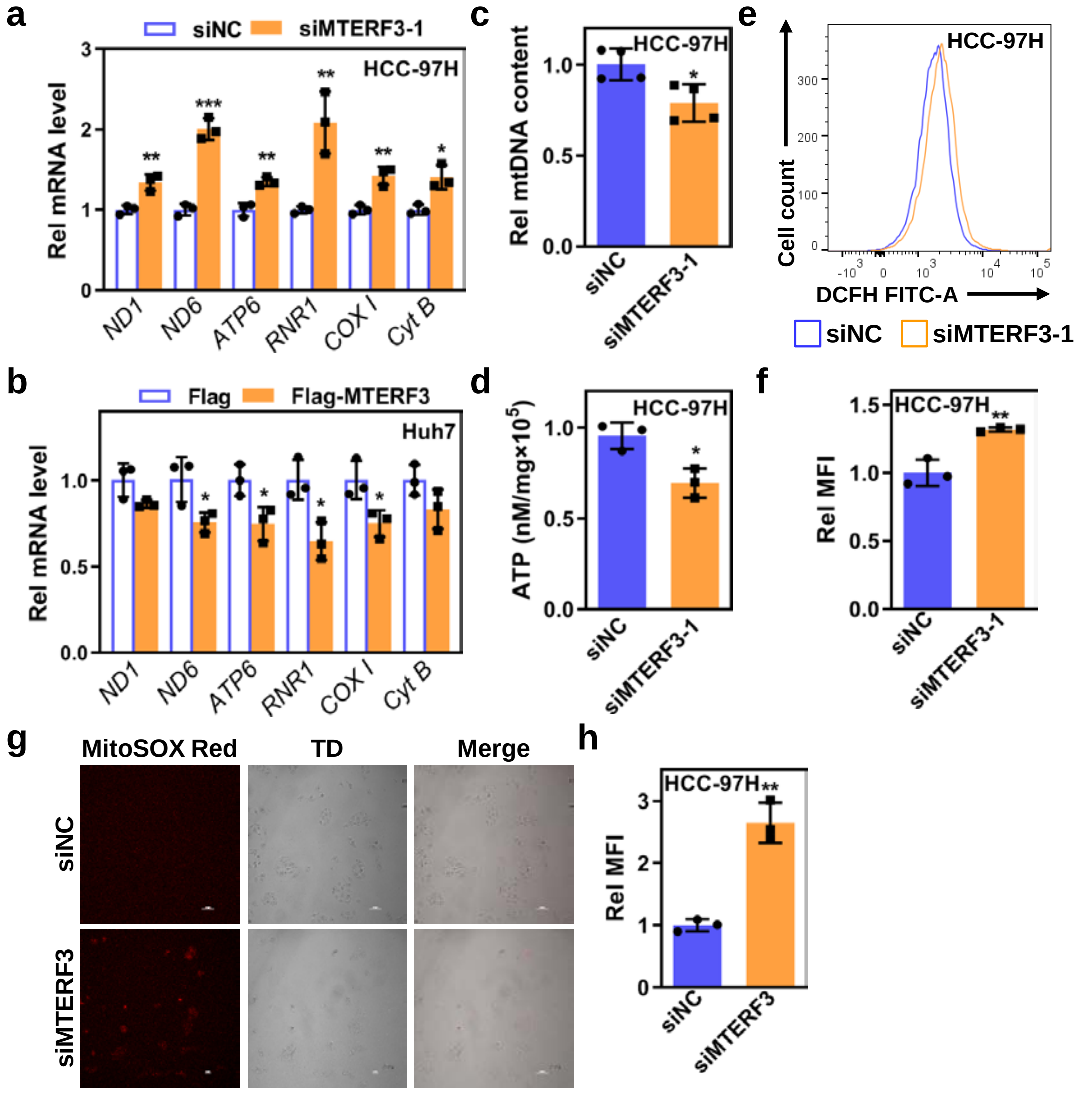

Supplementary Fig. 8 MTERF3 regulates mitochondrial function and ROS accumulation. (a) qRT-PCR to examine the transcription of mitochondrial genes in HCC-97H cells after transfected with indicated siRNA (n=3). (b) qRT-PCR to examine the transcription of mitochondrial genes in Huh7 cells with stably MTERF3 overexpression and controls (n=3). (c) qRT-PCR to examine the mtDNA content of HCC-97H after transfected with indicated siRNA (n=4). (d) ATP production was detected in HCC-97H after transfected with indicated siRNA (n=3). (e-f) The ROS level in HCC-97H cells transfected with indicated siRNA was analyzed, and the relative ROS level in (e) was calculated (n=3). (g) mitochondrial ROS production was analyzed in HCC-97H cells transfected with indicated siRNA by MitoSOX assay. (h) The relative ROS level in (g) was calculated (n=3). Data are shown as mean ± standard deviations of at least three independent experiments. Student's *t* test; **P*<0.05, ***P*<0.01 and ****P*<0.001.

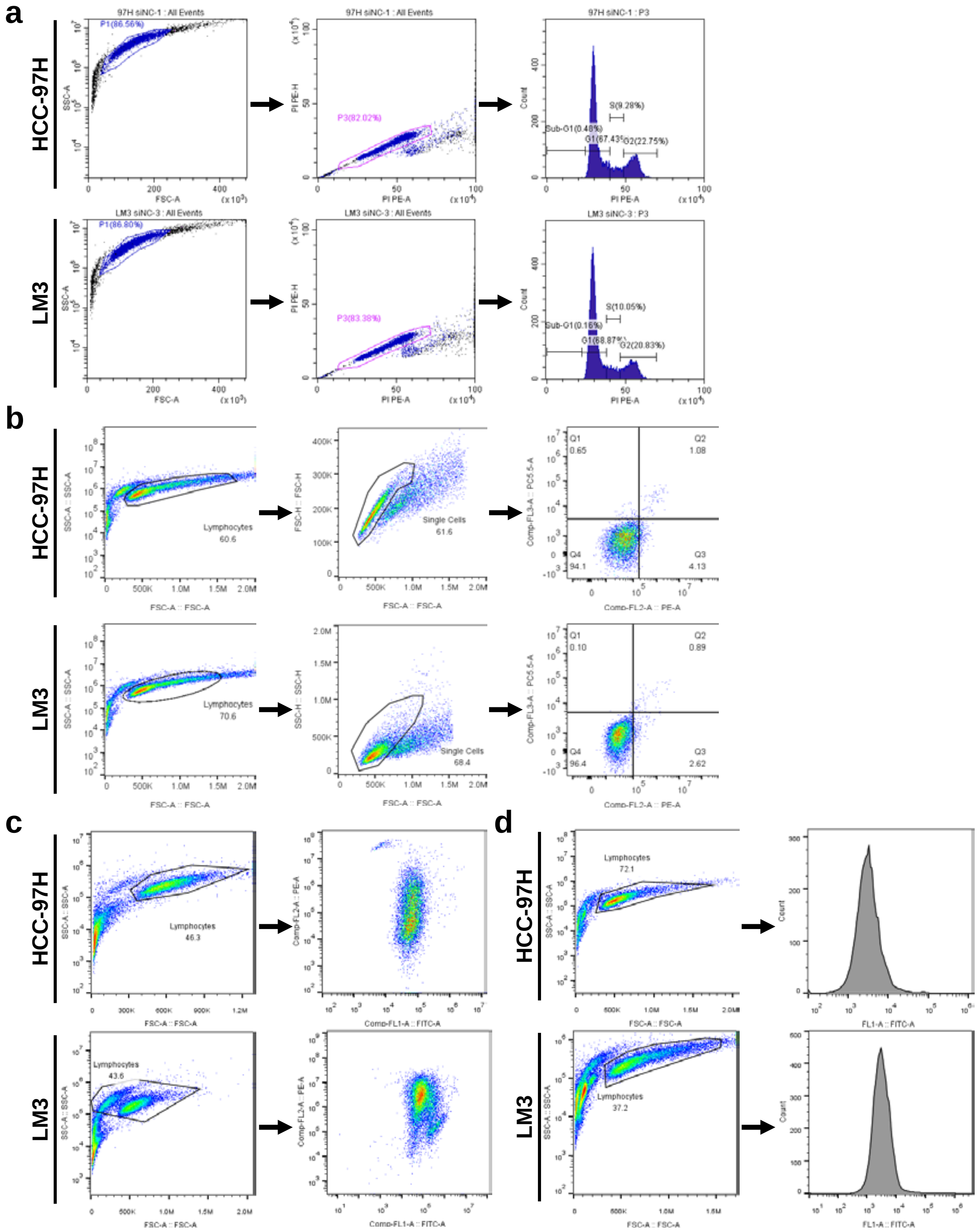

Supplementary Fig. 9 Gating strategy for flow cytometry in analysis of cell cycle distribution (a), apoptosis (b), MMP (c) and ROS (d) in HCC cells.

Supplemental Fig. 10: Unedited and uncropped Western blots of all the indicated figures in main figures and supplementary figures.

Fig. 1e

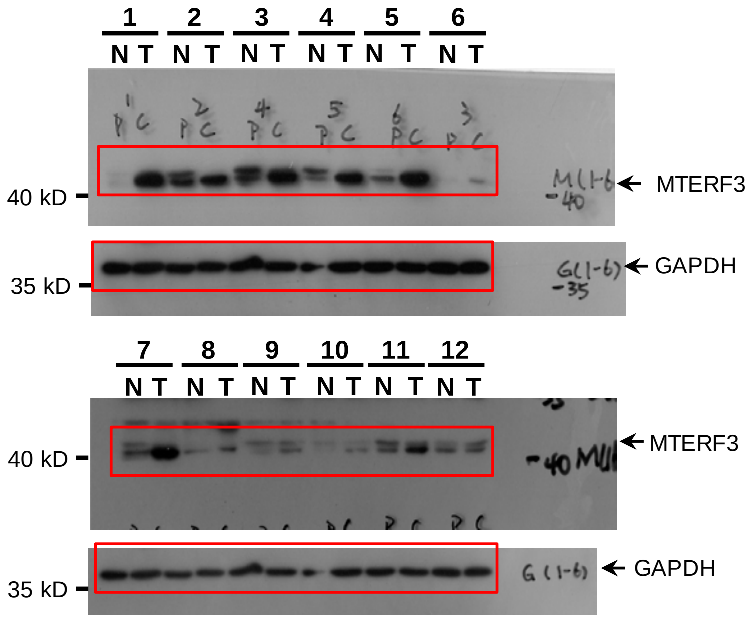

Fig. 2a

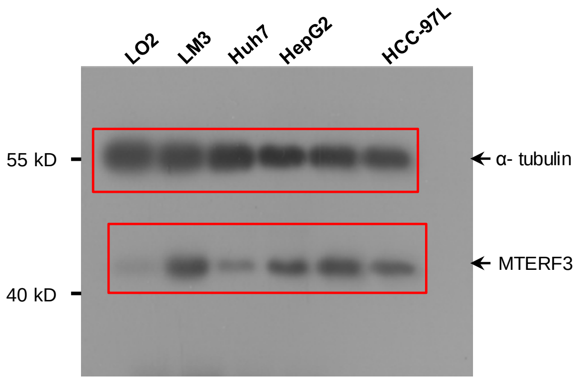

Fig. 2c

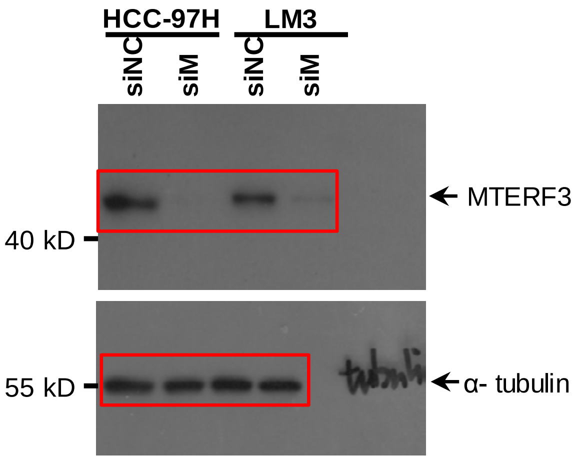

Fig. 2i

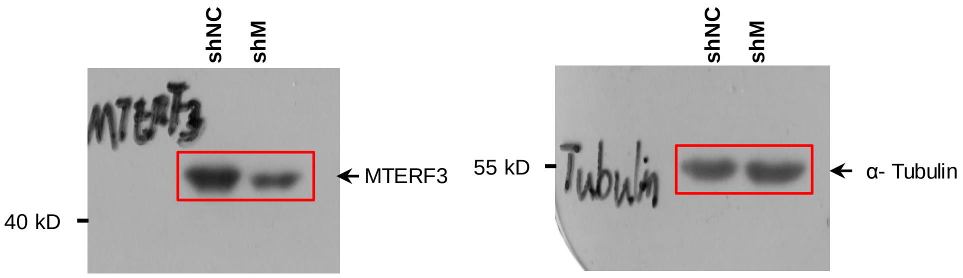

Fig. 3c

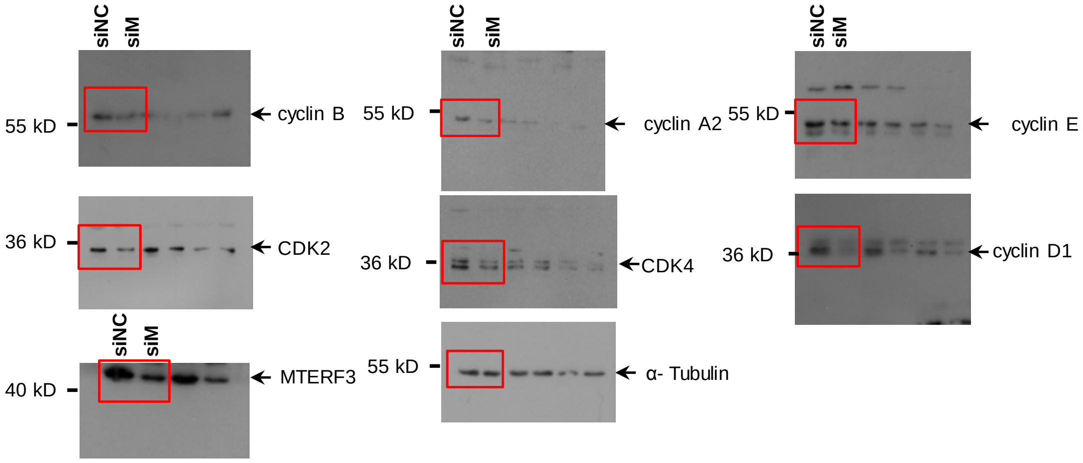

Fig. 3d

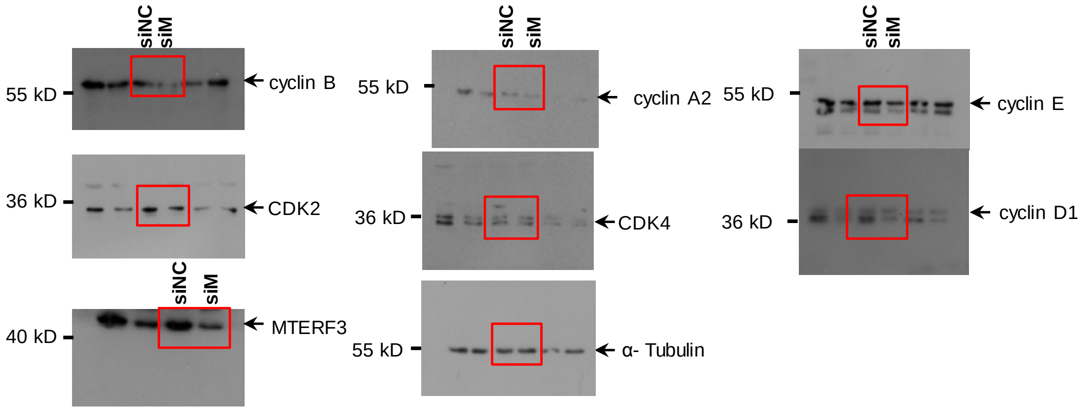

Fig. 4c

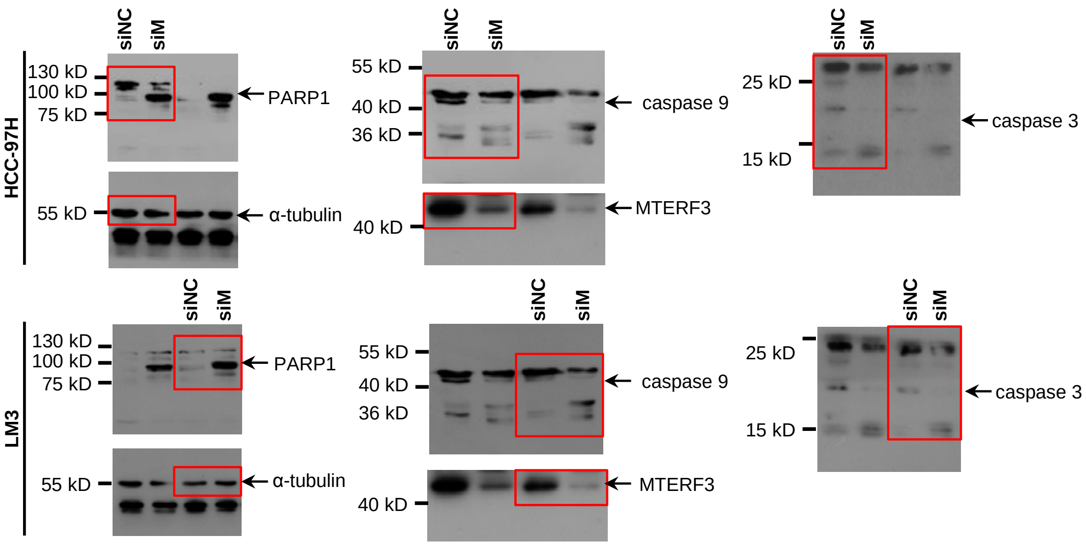
siNC
siM
HCC-97H
130 kD
100 kD
75 kD
PARP1
55 kD
α-tubulin
55 kD
40 kD
36 kD
caspase 9
40 kD
MTERF3
25 kD
15 kD
caspase 3
LM3
130 kD
100 kD
75 kD
PARP1
55 kD
α-tubulin
55 kD
40 kD
36 kD
caspase 9
40 kD
MTERF3
25 kD
15 kD
caspase 3

Fig. 4g

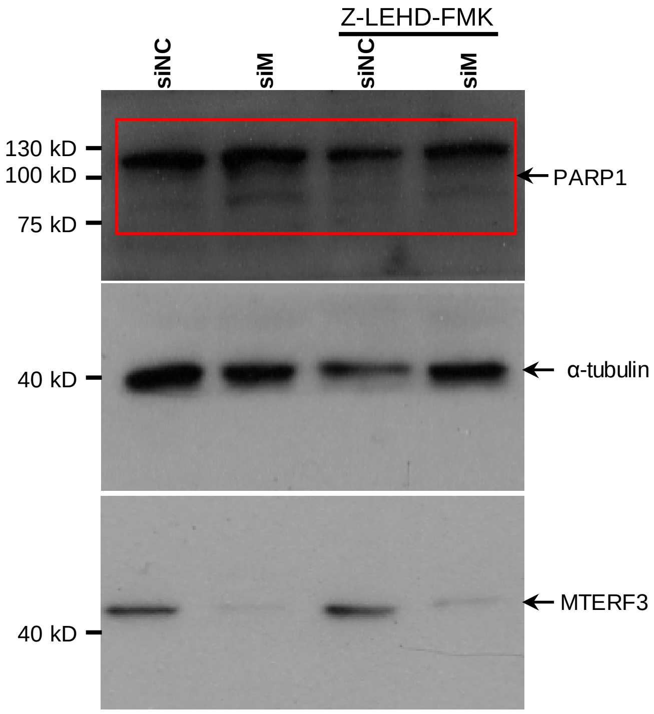
Z-LEHD-FMK
siNC
siM
siNC
siM
130 kD
100 kD
75 kD
PARP1
40 kD
α-tubulin
40 kD
MTERF3

Fig. 5a

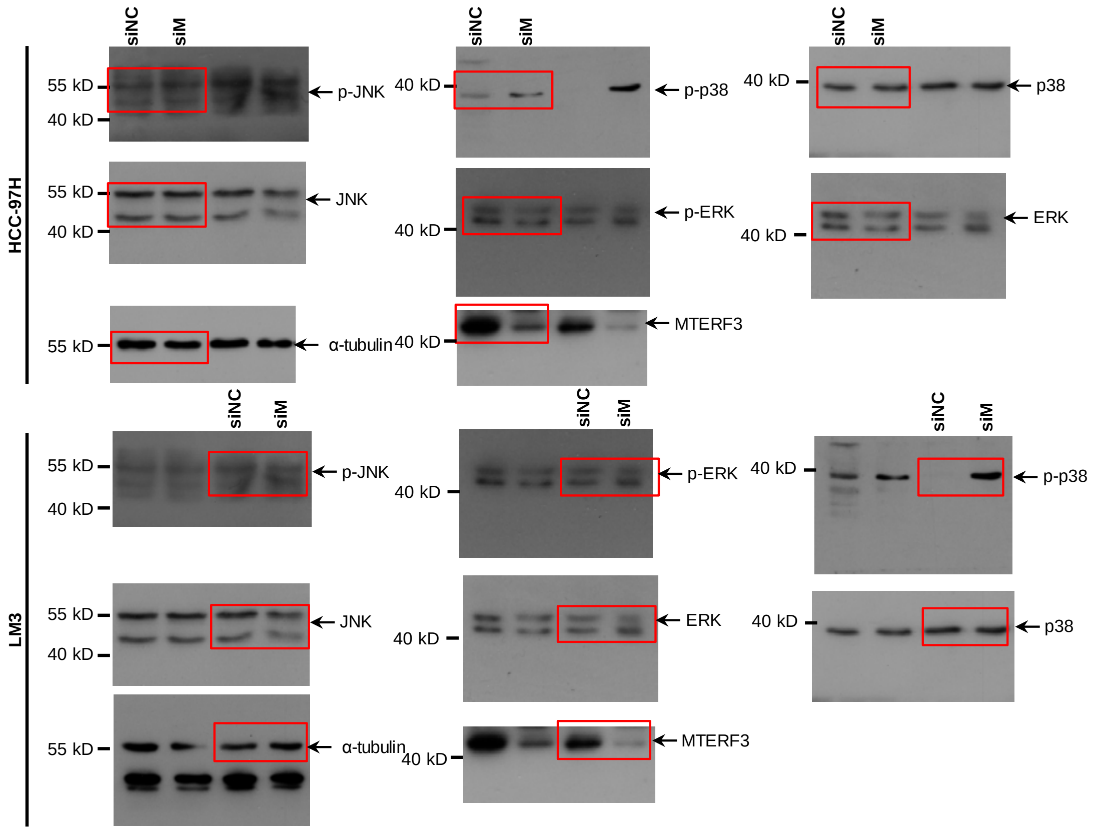

siNC
siM
HCC-97H
LM3
55 kD
40 kD
p-JNK
JNK
α-tubulin
p-p38
p-ERK
MTERF3
p38
ERK

Fig. 5b

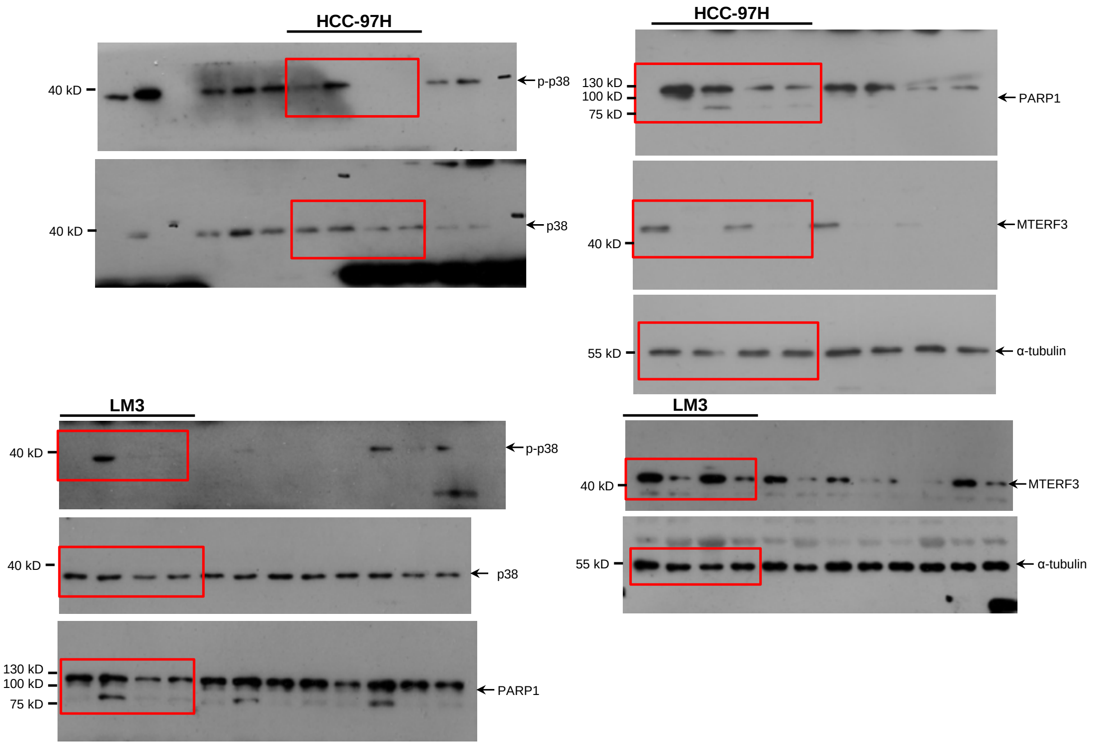

HCC-97H
LM3
40 kD
55 kD
130 kD
100 kD
75 kD
p-p38
p38
PARP1
MTERF3
α-tubulin

Fig. 5c

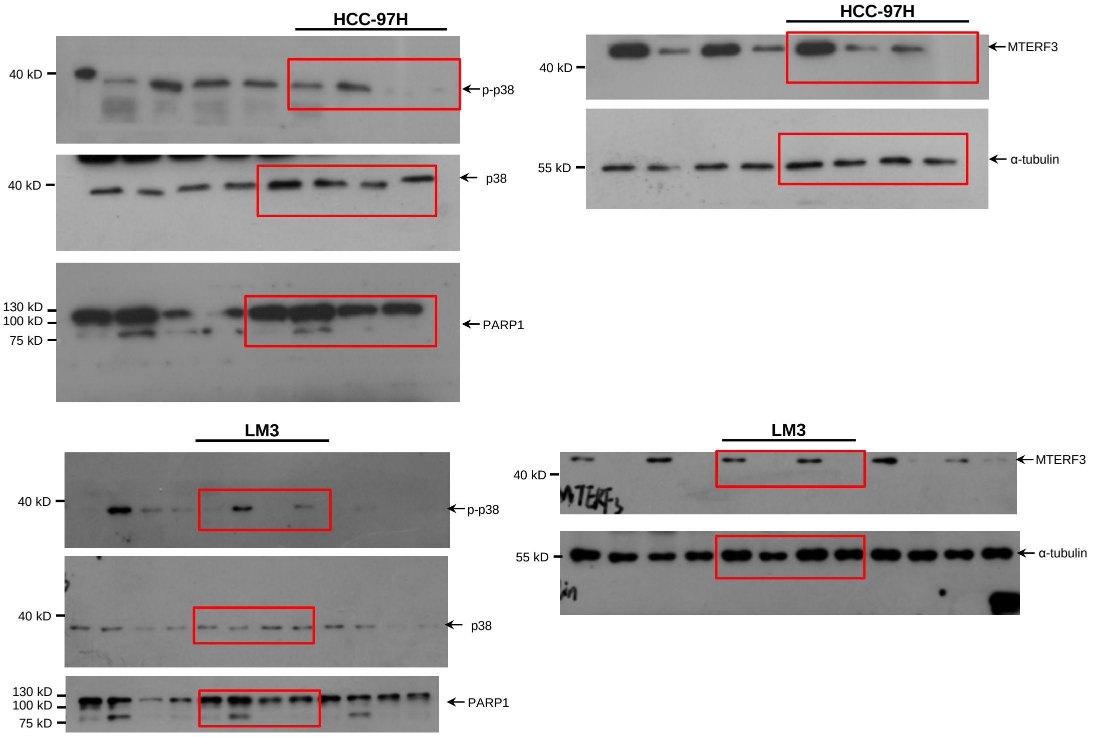
HCC-97H
40 kD
p-p38
p38
130 kD
100 kD
75 kD
PARP1
HCC-97H
MTERF3
55 kD
α-tubulin
LM3
p-p38
p38
PARP1
LM3
MTERF3
α-tubulin

Fig. 6j

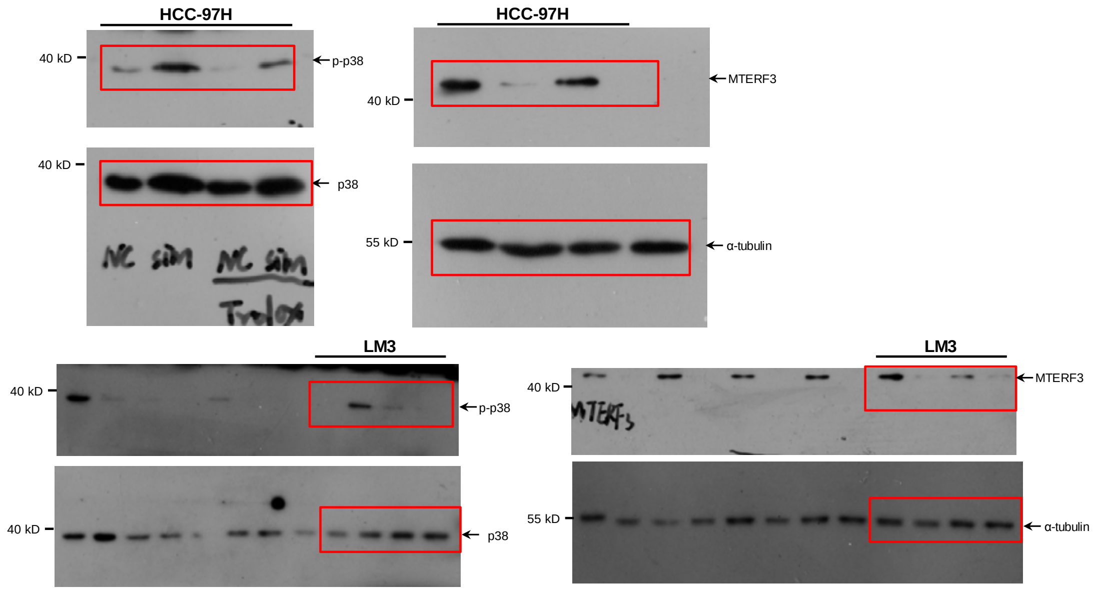
HCC-97H
40 kD
p-p38
p38
HCC-97H
MTERF3
55 kD
α-tubulin
LM3
p-p38
p38
LM3
MTERF3
α-tubulin

Supplementary Fig. 2a

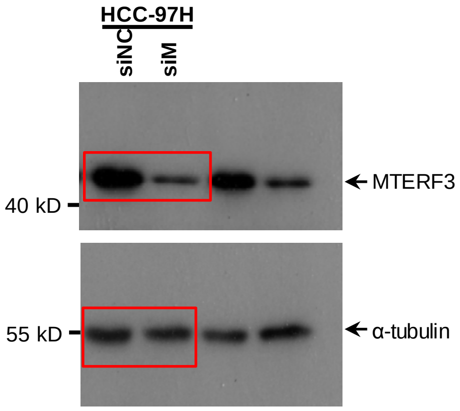

Supplementary Fig. 2b

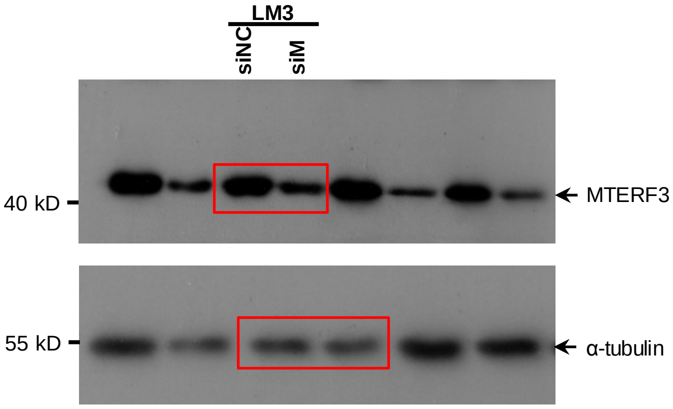

Supplementary Fig. 2e

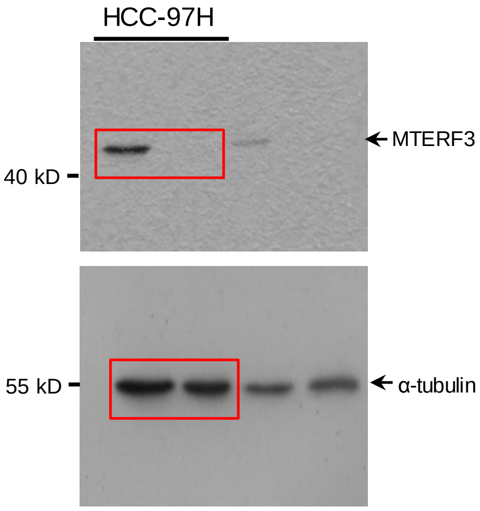

Supplementary Fig. 2f

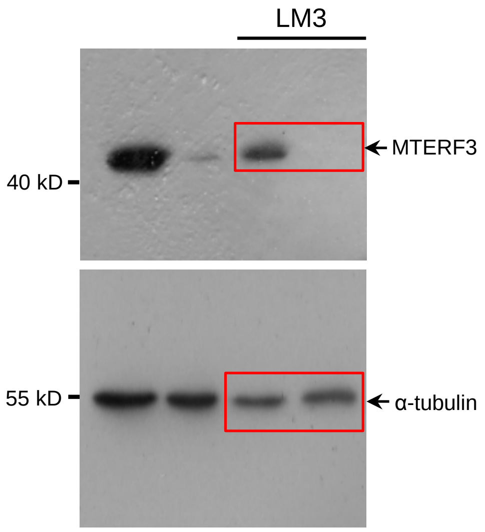

Supplementary Fig. 2i

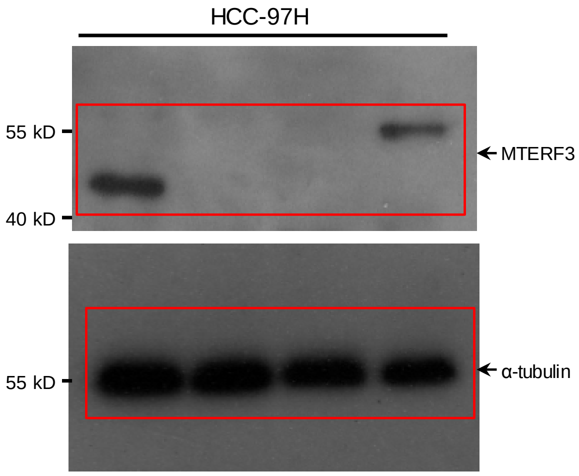

Supplementary Fig. 3a

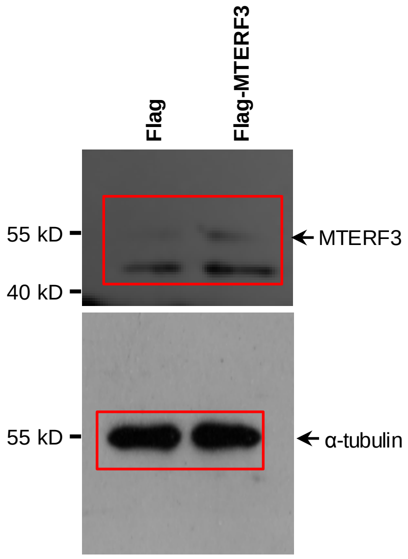

Supplementary Fig. 4g

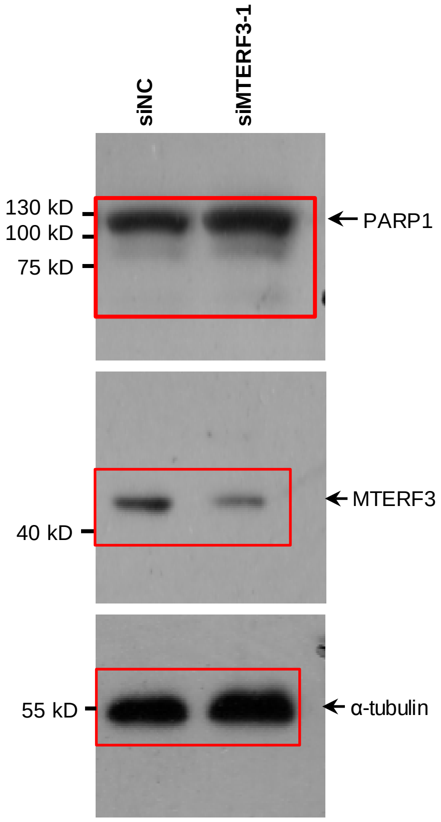

Supplementary Fig. 5c

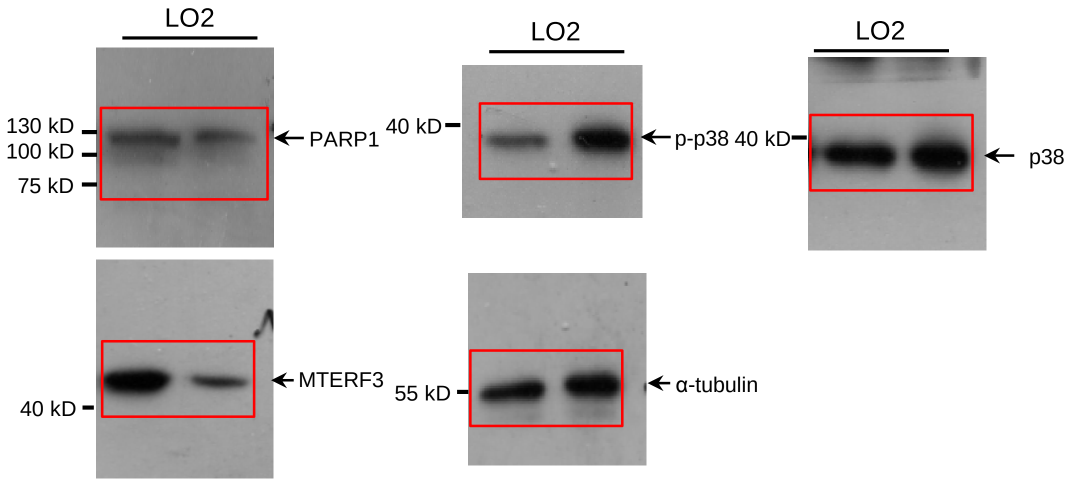

Supplementary Fig. 6a

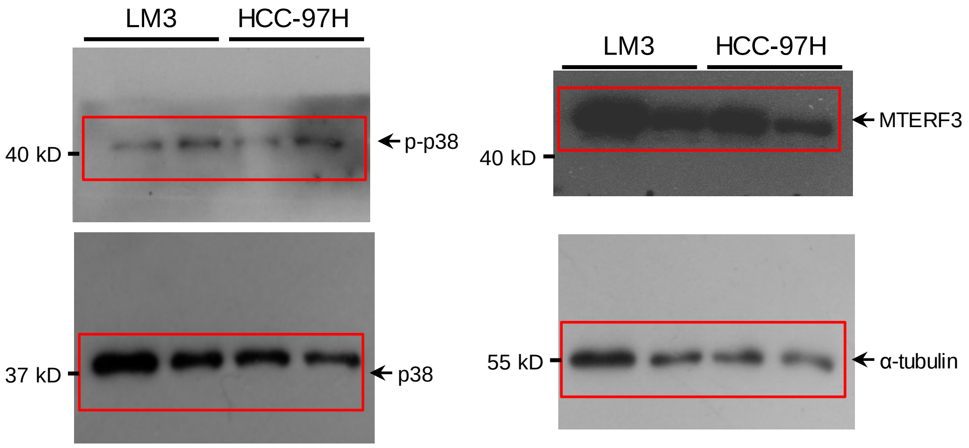

Supplementary Fig. 6b

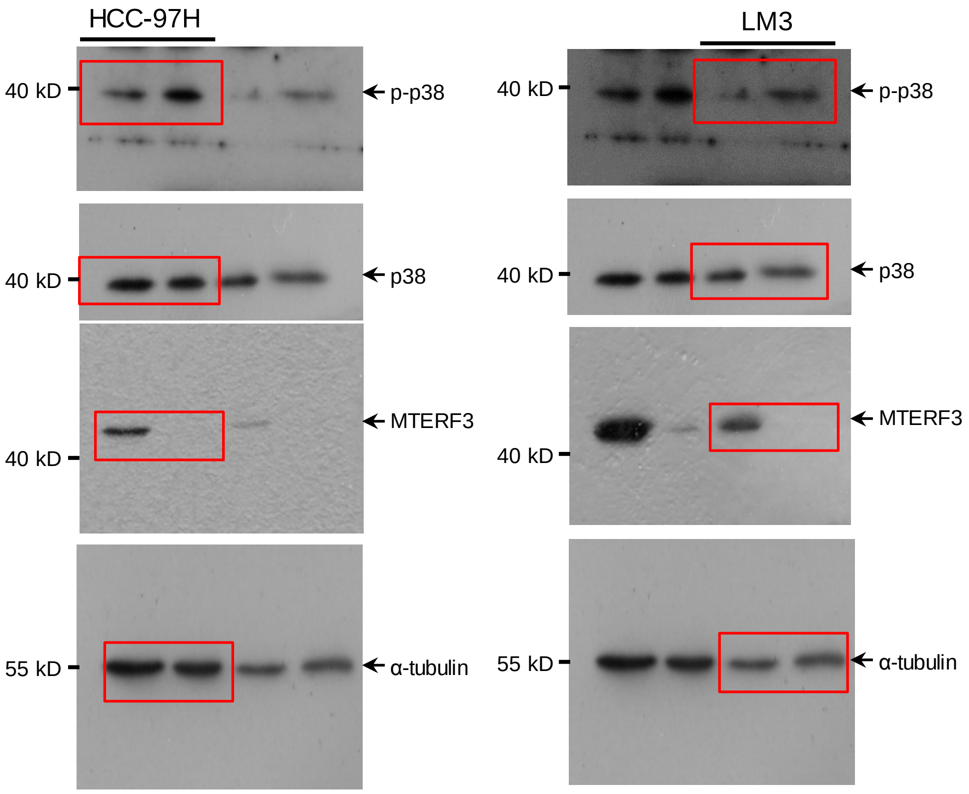

Supplementary Table 1 Antibodies and dilution ratio of relevant proteins used.

Antibodies	Source	Catalogue	Dilution
anti-MTERF3	Abcam	ab230232	1:1000
anti-α-tubulin	Sigma Aldrich	T5168	1:10000
anti-cyclin E	CST	#4129	1:1000
anti-cyclin B	Proteintech	55004-1-AP	1:1000
anti-cyclin A2	Proteintech	18202-1-AP	1:5000
anti-GAPDH	Proteintech	60004-I-Ig	1:10000
anti-CDK2	Saierbio	SRP00809	1:1000
anti-CDK4	Saierbio	SRP10419	1:1000
anti-cyclin D1	Proteintech	60186-1-lg	1:5000
anti-PAPP1	Proteintech	66520-1-lg	1:1000
anti-caspase 9	CST	#9508	1:1000
anti-caspase 3	CST	#14220	1:1000
anti-p-p38	CST	#4511	1:1000
anti-p38	CST	#8690	1:1000
anti-p-ERK	CST	#4370	1:1000
anti-ERK	CST	#9102	1:1000
anti-p-JNK	CST	#9255	1:1000
anti-JNK	CST	JNK	1:1000
Goat anti-Rabbit HRP	Affinity Biosciences	S0001	1:15000
Goat anti-Mouse HRP	Affinity Biosciences	S0002	1:15000
