# Supplementary material for: Suppressing MTERF3 inhibits proliferation of human hepatocellular carcinoma via ROS-mediated p38 MAPK activation
Source: Commun Biol. 2024 Jan 5;7:18. doi: 10.1038/s42003-023-05664-7 (PMC10767110; doi:10.1038/s42003-023-05664-7)
Supplement: Supplementary file 5 — Reporting Summary [file 42003_2023_5664_MOESM5_ESM.pdf]

nature portfolio

Corresponding author(s): Zhixiong Dong

Last updated by author(s): Nov 17, 2023

Reporting Summary

Nature Portfolio wishes to improve the reproducibility of the work that we publish. This form provides structure for consistency and transparency in reporting. For further information on Nature Portfolio policies, see our Editorial Policies and the Editorial Policy Checklist.

Statistics

For all statistical analyses, confirm that the following items are present in the figure legend, table legend, main text, or Methods section.

n/a	Confirmed	
☐	☒	The exact sample size (*n*) for each experimental group/condition, given as a discrete number and unit of measurement
☐	☒	A statement on whether measurements were taken from distinct samples or whether the same sample was measured repeatedly
☐	☒	The statistical test(s) used AND whether they are one- or two-sided<br>*Only common tests should be described solely by name; describe more complex techniques in the Methods section.*
☐	☒	A description of all covariates tested
☐	☒	A description of any assumptions or corrections, such as tests of normality and adjustment for multiple comparisons
☐	☒	A full description of the statistical parameters including central tendency (e.g. means) or other basic estimates (e.g. regression coefficient) AND variation (e.g. standard deviation) or associated estimates of uncertainty (e.g. confidence intervals)
☐	☒	For null hypothesis testing, the test statistic (e.g. *F*, *t*, *r*) with confidence intervals, effect sizes, degrees of freedom and *P* value noted<br>*Give P values as exact values whenever suitable.*
☒	☐	For Bayesian analysis, information on the choice of priors and Markov chain Monte Carlo settings
☒	☐	For hierarchical and complex designs, identification of the appropriate level for tests and full reporting of outcomes
☒	☐	Estimates of effect sizes (e.g. Cohen's *d*, Pearson's *r*), indicating how they were calculated

Our web collection on statistics for biologists contains articles on many of the points above.

Software and code

Policy information about availability of computer code

Data collection	No software was used
Data analysis	Graphpad Prism 7 was used to analyze the statistical significance. Image J software was used to quantified the density of western blot and the florescent intensity of mitoROS. Image J software was also used for counting the colony number.

For manuscripts utilizing custom algorithms or software that are central to the research but not yet described in published literature, software must be made available to editors and reviewers. We strongly encourage code deposition in a community repository (e.g. GitHub). See the Nature Portfolio guidelines for submitting code & software for further information.

Data

Policy information about availability of data

All manuscripts must include a data availability statement. This statement should provide the following information, where applicable:

- Accession codes, unique identifiers, or web links for publicly available datasets
- A description of any restrictions on data availability
- For clinical datasets or third party data, please ensure that the statement adheres to our policy

The datasets generated in the current study are available from the corresponding authors on reasonable request.

Human research participants

Policy information about studies involving human research participants and Sex and Gender in Research.

Reporting on sex and gender	Female and male patients were randomly enrolled.
Population characteristics	Patients who were diagnosed primary HCC and underwent standard surgical treatment.
Recruitment	All of patients with primary HCC were underwent standard surgical treatment and histopathological examination in the Affiliated Hospital of Wenzhou Medical University (Wenzhou, China). Each sample was attached to a confirmed pathological diagnosis and was staged according to the 8th edition of the American Joint Committee on Cancer (AJCC) cancer staging manual.
Ethics oversight	Experiment were conducted with the approval of the Institutional Research Ethics Committee of Wenzhou Medical University

Note that full information on the approval of the study protocol must also be provided in the manuscript.

Field-specific reporting

Please select the one below that is the best fit for your research. If you are not sure, read the appropriate sections before making your selection.

☒ Life sciences ☐ Behavioural & social sciences ☐ Ecological, evolutionary & environmental sciences

For a reference copy of the document with all sections, see nature.com/documents/nr-reporting-summary-flat.pdf

Life sciences study design

All studies must disclose on these points even when the disclosure is negative.

Sample size	the sample size was satisfied the requirement of the related statistical methods.
Data exclusions	No data was excluded in our study.
Replication	Animal study were performed with biological replicates. The other experiments were performed at least three times.
Randomization	All of samples collection are randomized. Mice were randomized to treatment groups.
Blinding	All quantifications of the raw data were done blind to conditions.

Reporting for specific materials, systems and methods

We require information from authors about some types of materials, experimental systems and methods used in many studies. Here, indicate whether each material, system or method listed is relevant to your study. If you are not sure if a list item applies to your research, read the appropriate section before selecting a response.

Materials & experimental systems

n/a	Involved in the study	
☐	☒	Antibodies
☐	☒	Eukaryotic cell lines
☒	☐	Palaeontology and archaeology
☐	☒	Animals and other organisms
☒	☐	Clinical data
☒	☐	Dual use research of concern

Methods

n/a	Involved in the study	
☒	☐	ChIP-seq
☐	☒	Flow cytometry
☒	☐	MRI-based neuroimaging

Antibodies

Antibodies used	mouse anti-α-tubulin (T5168, Sigma Aldrich, 1:10000), Rabbit anti-MTERF3 (ab230232, Abcam, 1:1000), Rabbit anti-p-p38 (#4511, CST, 1:1000), Rabbit anti-p38 (#8690, CST, 1:1000), Rabbit anti-p-ERK (#4370, CST, 1:1000), Rabbit anti-ERK (#9102, CST, 1:1000), Rabbit anti-p-JNK (#9255, CST, 1:1000), Rabbit anti-JNK (#9252, CST, 1:1000), Rabbit anti-PARP1 (66520-1-lg, Proteintech, 1:1000), Rabbit anti-cleaved caspase 3 (#9661, CST, 1:1000), mouse anti-cyclin B (55004-1-AP, 1:5000), mouse anti-cyclin A (18202-1-AP, 1:5000), Rabbit anti-cyclin E (#4129, CST, 1:1000), Rabbit anti-cyclin D (60186-1-lg, Proteintech, 1:5000), Rabbit anti-CDK2

	(SRP00809, Saierbio, 1:1000), Rabbit anti-CDK4 (SRP10419, 1:1000). Peroxidase-conjugated anti-mouse IgG (S0002, Affinity Biosciences, 1:14000) and anti-rabbit IgG (S0001, Affinity Biosciences, 1:14000).
Validation	All antibodies are widely used , well validated commercial products. Details can be referred on the manufactures' websites.

Eukaryotic cell lines

Policy information about cell lines and Sex and Gender in Research

Cell line source(s)	Human HCC cell lines, including HCC-97H, HCC-97L, LM3, Huh7 and HepG2, and a normal hepatocytes cells LO2 were provided by Dr. Liang Xu (Wenzhou Medical University).
Authentication	Yes
Mycoplasma contamination	All of cells are negative for mycoplasma contamination
Commonly misidentified lines (See ICLAC register)	N/A

Animals and other research organisms

Policy information about studies involving animals; ARRIVE guidelines recommended for reporting animal research, and Sex and Gender in Research

Laboratory animals	BALB/c female nude mice, 3-4 weeks old.
Wild animals	The study did not involved wild animals.
Reporting on sex	10 female nude mice were used in this study.
Field-collected samples	Body weight and tumor volume were monitored every three days in SPF house. Lastly, tumor tissues were collected from euthanasian mice.
Ethics oversight	The animal experiments were approved by the Ethics Committee for Laboratory Animals of the Wenzhou Medical University.

Note that full information on the approval of the study protocol must also be provided in the manuscript.

Flow Cytometry

Plots

Confirm that:

☒ The axis labels state the marker and fluorochrome used (e.g. CD4-FITC).

☒ The axis scales are clearly visible. Include numbers along axes only for bottom left plot of group (a 'group' is an analysis of identical markers).

☒ All plots are contour plots with outliers or pseudocolor plots.

☒ A numerical value for number of cells or percentage (with statistics) is provided.

Methodology

Sample preparation	HCC-97H or LM3 cells were transfected with siNC or siMTERF3 for 72 h, and the cells were fixed or labeled with Annexin V/7-AAD kit, JC-1 assay kit or Reactive Oxygen Species Assay Kit according to the instructions.
Instrument	CytoFLEX (Beckman Coulter, USA)
Software	CytoFLEX software was used to collect cells. CytExpert was used to analyze the cell cycle distribution. FlowJo_v10.6.2 software was used to analyze apoptosis, mitochondrial membrane potential and ROS.
Cell population abundance	At least 10000 cells were collected for analysis.
Gating strategy	The gate was set to capture the majority of HCC cells.

☒ Tick this box to confirm that a figure exemplifying the gating strategy is provided in the Supplementary Information.
